# Supplementary material for: Epidemiological description of Marburg virus disease outbreak in Kagera region, Northwestern Tanzania
Source: PLoS One. 2024 Sep 5;19(9):e0309762. doi: 10.1371/journal.pone.0309762 (PMC11376503; doi:10.1371/journal.pone.0309762)
Supplement: Attachment — Submitted filename: Response to reviewers comments.docx [file pone.0309762.s002.docx]

**Dear Editor,**

We would like to thank the editor and reviewers for their helpful feedback on our manuscript, **"Epidemiological description of Marburg virus disease outbreak in Kagera region, Northwestern Tanzania".** Their comments have significantly improved the quality of our work, and we have carefully considered each suggestion in the revised version of the paper. We are confident that we have adequately addressed the raised concerns and that the manuscript is now ready for submission.

Warm regards,

**Dr. George Mrema**

**Editor**

**Comments/concerns/suggestions by editor:**

1. Please verify the number of samples listed under "Type of exposure" in Table 1. Is it indeed 309, or should it be 306?

**Response to the editor:** We appreciate editor’s observations. Typo has been corrected (Table 1).

2. Ensure that scales are provided for each map in the figures.

**Response to the editor:** We appreciate editor’s comment. We are grateful for the editor's suggestions. Every map that has been created contains a unique legend (Figure 2 and 3).

3. In Figure 1, please provide an explanation of the numerical values depicted.

**Response to the editor:** We thank the editor for this comment. The number indicated correlate to cases reported in this outbreak. However, after careful consideration, we have decided to exclude the numbers shown in Figure 1 as we do not think their omission will change the significance of the epi curve (Fig 1).

**Reviewer No. 1**

**Comments/concerns/suggestions by reviewer 1:**

1. Sentence beginning line 63 to 64 - The etiologies of MVD are RNA viruses………. is incomplete.

**Response to the reviewer:** We appreciate the reviewer's suggestions. We have rephrased and completed the sentence, making it clear with a logical flow (line 62-64).

2. Sentence beginning line 74 to 77 - The SUDV recorded 164 cases…….. should be rephrased, e.g. There were 164 cases of EVD (142 confirmed and 22 probable) and….

**Response to the reviewer:** Thank you so much for the comment. We rephrased the sentence to enhance the connection between sentences and make it clearer (line 72-84).

3. Line 77: Kagera Region - region is capitalised, but throughout the manuscript it is all lowercase

**Response to the reviewer:** We thank the reviewer for pointing out this. The word "region" was unnecessarily capitalized, so it has been changed to lowercase to match the rest of the manuscript (line 77).

4. Line 158: The last sentence is incomplete.

**Response to the reviewer:** We appreciate the reviewer's observation. We have omitted the incomplete sentence that was not supposed to be included (line 158).

5. Line 161: “information about on…”.

**Response to the reviewer:** We appreciate the observation. The word "on" has been removed, and the sentence now has a clearer flow (line 161).

6. Line 213: personal (case 5).

**Response to the reviewer:** We thank the reviewer for the comment. We have rephrased that segment to make it easier to understand (line 219).

7. Sentence beginning line 227: On March 13, 2023, her sister-in-law (Case 4), who was the mother of the index case, visited her, but she also reported feeling unwell.

**Response to the reviewer:** We thank the reviewer for pointing out this. We have rephrased our statement and its now clear for the readers (line 231-237).

8. Line 310: here and in other parts of the manuscript fig should be capitalized. Also, if it is in text, it should not be bold.

**Response to the reviewer:** Thank you so much for the comment. We have adjusted the names of all the figures to align with your description and instructions provided in the journal (line 192, 313 & 328-329).

9. Line 322: either should be used with or. In this case either can be removed

**Response to the reviewer:** Thank you for this suggestion. We have removed the word either as suggested (line 325).

10. Line 325: “And the majority”

**Response to the reviewer:** We appreciate for this observation. We rephrased the sentence to enhance the connection between sentences and make it clearer (Line 327-329).

11. Line 394: there are 2 full stops.

**Response to the reviewer:** Thank you so much for the comment. One full stop has been deleted (line 396).

12. Line 404: the sentence “To strengthen early detection and reporting at all levels.” Seems to be missing something at the beginning

**Response to the reviewer:** We thank the reviewer for pointing out this. We have added words at the beginning, it is now clear to readers (line 406).

13. There have been changes to filovirus taxonomy. The authors should follow the International Committee for Taxonomy of Viruses instructions for the taxonomy of Filoviruses. (Biedenkopf, N.; Bukreyev, A.; Chandran, K.; Di Paola, N.; Formenty, P. B. H.; Griffiths, A.; Hume, A. J.; Mühlberger, E.; Netesov, S. V.; Palacios, G.; Pawęska, J. T.; Smither, S.; Takada, A.; Wahl, V.; Kuhn, J. H., Renaming of genera Ebolavirus and Marburgvirus to Orthoebolavirus and Orthomarburgvirus, respectively, and introduction of binomial species names within family Filoviridae. Arch Virol 2023, 168, (8), 220. <https://doi.org/10.1007/s00705-023-05834-2>.)

**Response to the reviewer:** Thank you for your comment. We have introduced the new terminology in the background to inform readers about the updated naming of MVD (line 61).

**Other Comments**

14. Line 73: I suggest it read: “On September 20th, 2022, an outbreak of Ebola virus disease (EVD) caused by Sudan Virus (SUDV) was declared by the government of Uganda in Mubende district Western Uganda.” This will also allow for the definition of EVD that is in line 83, as in the manuscript it is introduced for the first time, but is not written out in full.

**Response to the reviewer:** Thank you so much for the suggestions. The entire paragraph has been rewritten, and the term Ebola Virus Disease is now spelled out in full since it is being used for the first time (line 72-84).

15. Line 77: I think it is important to indicate that Mutukula town is in Tanzania. This means that line 78 can end “90 km (55 miles) from Mutukula town.” And delete “on the Kagera region (Tanzania), border.”.

**Response to the reviewer:** Thank you so much for the comment. We have taken your suggestion into consideration and made the necessary improvements to our statement as per your recommendation (line 75-77).

16. Line 83: EVD is introduced for the first time, but is not written out in full. I think it would be better to add a sentence indicating that EVD and MVD have similar presentation.

**Response to the reviewer:** We appreciate the suggestion. We have included a sentence that describes the similarity between EVD and MVD, linking it to the EVD outbreak in Uganda and how it helped the country respond to the MVD outbreak (line 77-84).

17. Line 188: refers the index. I presume this should read the probable index case. Here and throughout the manuscript, it would be better to refer to this case as probable index case and not index case.

**Response to the reviewer:** We appreciate the reviewer's suggestion. We have changed the word “index case” to “probable index case” in the whole document as suggested

18. Line 204: this is interesting past medical history of vomiting blood. Was there any indication on the timing? Was it the recent past? It may be better to indicate.

**Response to the reviewer:** Thank you so much for the comment. We have indicated the timing of his past episodes of vomiting blood. (line 207-210).

19. Case 7: I think it would be better to indicate form the 1st sentence that Case & treated the index case. This gives a clearer epidemiological picture. Therefore “Index case on 28th February” should move from line 280 to a more appropriate line.

**Response to the reviewer:** We thank the reviewer for pointing out this. We have rephrased our explanation and information on treatment of probable index case and other cases has been moved at the beginning, it is now clear to readers (line 285-288).

20. Line 349: it should be clear that the laboratory confirmation was not from the probable index case

**Response to the reviewer:** We appreciate the reviewer's suggestions. We have revised our statement to make it clear to the reader that the probable index case was not confirmed through laboratory testing (line 350-351).

21. Lines 357-360: in this section, the outbreak year has only been given for Uganda. Is there a specific reason? If not, it is better to be consistent and indicate the outbreak year for all or none

**Response to the reviewer:** Thank you so much for the comment. Due to the multiple outbreaks in Uganda occurring in different years, we have included specific years in each outbreak mentioned to clarify which outbreak we are referring to. Furthermore, we have included the year for all other mentioned outbreaks to maintain consistency (line 359-362).

22. Lines 363-367: it has been documented in literature that ignorance of health care workers about the disease is one of the reasons there is a delay in detection of an outbreak of EVD/MVD, particularly in areas where no previous outbreak has occurred. It would be good to make reference to this

**Response to the reviewer:** We thank the reviewer for the comment. We have included a paragraph that explains how delays in the diagnosis of MVD can occur in a healthcare setting and the possible reasons for these delays (line 365-370).

23. Lines 368-373: the basic reproduction number for Marburg virus is relatively low. It would be good to make a comment on this aspect.

**Response to the reviewer:** We thank the reviewer for the comment. We have included details on the transmission nature of MVD to explain why only a small number of contacts became cases (line 372-375).

24. Line 374-375: best to put (VHF) after viral hemorrhagic fever as it is the first time it is appearing in the manuscript

**Response to the reviewer:** Thank you for this suggestion. The abbreviation has been added in line 82 when we rewrote that section and kept the abbreviation (line 376-377).

25. Line 388: It would be better to replace SUDV with “the EVD outbreak”

**Response to the reviewer:** The comments have been noted, and the term SUDV has been replaced with EVD outbreak (line 390).

**Reviewer No. 2**

**Comments/concerns/suggestions by reviewer 2:**

1. line70: the marburg outbreak in Equatorial Guinea occured in 2023...not 2021.

**Response to the reviewer:** We thank the reviewer for pointing out this. The typo has been corrected (line 70).

2. line 37-38. What does the sentence "laboratory testing used altona diagnostics realstart filovirus screen RTPCR with at least 2 tests in two separate laboratories" mean? do you mean that the sample sample had to be tested using the same assay in two geographically separate laboratories? if so, why?

**Response to the reviewer:** Thank you so much for the comment. The samples were tested in different locations as a means of confirmation, as this was the first outbreak of that type to happen in the country. The Ministry of Health had to ensure that all the results are of high quality (line 137-140).

3. line: 156: I think there might be a typo....currently it says that "any contacts who developed symptoms were re-classified as a case....i think you mean "suspect"

**Response to the reviewer:** We thank the reviewer for the comment. In this paragraph, we meant that those people who are being monitored as contacts, as soon as they develop symptoms, they will become suspects, hence their status will change to cases. Additionally, We have changed the term “case” to “suspected case.” (line 158-159).

4. Line 158: the last sentence is incomplete.

**Response to the reviewer:** Thank you, the incomplete sentence has been resolved (line 160).

5. Line 163: did the reach out to close family members of the individuals involved occur during the outbreak? or afterwards?

**Response to the reviewer:** We thank the reviewer for the comment. we have revised our statement to better convey that the information was gathered post-outbreak during data extraction (line 165-167).

6. Description and epidemiologic linkage of MVD cases: it might be best to summarize the key findings for each patient and place the detailed summaries in the appendix? Also, it would be helpful to provide additional epi details on case 1 (such as his occupation and details on how he might have been infected...some of this information is currently in the discussion section)

**Response to the reviewer:** We are grateful to the reviewer for the suggestion. We have included updated information on occupation and the potential source of the outbreak, which was not discovered until the end of the outbreak (lines 202-206). After consulting with our co-authors, we have decided against moving the information on cases to the appendix. This is because a significant portion of the paper relies on the information about the cases and their connections, and relocating them to the appendix would diminish the importance of the article.

7. line 322-323: what is the difference between family members and relatives?

**Response to the reviewer:** Thank you so much for the comment. After careful consideration, we have decided to omit the word "relative" and instead use "family members" since they are essentially synonymous (line 325-326, Table 1).

8. line 359: would suggest not including the CFR for the Netherlands as it was just 1 patient.

**Response to the reviewer:** Thank you for this suggestion. Netherland MVD CFR has been from comparison (line 361-362).

9. Fig 2: not sure if this figure is necessary

**Response to the reviewer:** We appreciate the reviewer's suggestions. Fig 2 has been removed as suggested.
