# Supplementary material for: Epidemiological description of Marburg virus disease outbreak in Kagera region, Northwestern Tanzania
Source: PLoS One. 2024 Sep 5;19(9):e0309762. doi: 10.1371/journal.pone.0309762 (PMC11376503; doi:10.1371/journal.pone.0309762)
Supplement: Attachment — Submitted filename: Response to reviewers comments.docx [file pone.0309762.s003.docx]

**Dear Editor,**

We would like to express our gratitude to the editor and reviewers for their valuable feedback on our manuscript titled **"Epidemiological description of Marburg virus disease outbreak in Kagera region, Northwestern Tanzania".** We have taken into account all of their suggestions and believe that our manuscript has been greatly improved as a result. We are confident that we have addressed all concerns and the revised manuscript is now ready to be re submitted.

Warm regards,

**Dr. George Mrema**

**Editor**

**Comments/concerns/suggestions by editor:**

1. Regarding my comment 2, "Ensure that scales are provided for each map in the figures," I can accept the current figures even though map scales are not provided (of course, it is better to include them since not all readers are familiar with Tanzania's geology). However, the shapefiles are not available from the URL provided in the legend. Please check the URL again

**Response to the editor:** We appreciate editor’s comment. Scale has been included in all maps and URL has been updated in the manuscript (Figure 2 and 3).

**Reviewer No. 1**

**Comments/concerns/suggestions by reviewer 1:**

1. My recommendations on the nomenclature of Filoviruses have not been addressed adequately. The lines 61 to 64 need to be rewritten to convey the correct nomenclature. The authors should follow the International Committee for Taxonomy of Viruses instructions for the taxonomy of Filoviruses. (Biedenkopf, N.; Bukreyev, A.; Chandran, K.; Di Paola, N.; Formenty, P. B. H.; Griffiths, A.; Hume, A. J.; Mühlberger, E.; Netesov, S. V.; Palacios, G.; Pawęska, J. T.; Smither, S.; Takada, A.; Wahl, V.; Kuhn, J. H., Renaming of genera Ebolavirus and Marburgvirus to Orthoebolavirus and Orthomarburgvirus, respectively, and introduction of binomial species names within family Filoviridae. Arch Virol 2023, 168, (8), 220. https://doi.org/10.1007/s00705-023-05834-2.).

**Response to the reviewer:** We appreciate reviewer's suggestions. We have incorporated the updated taxonomy of the Filoviridae family into the manuscript (Line 62-69 & Line 77-89).

2. Line 61: Marburg virus disease has not been renamed as Orthomarburgvirus. Marburg virus disease is caused by the orthomarburgviruses Marburg virus and Ravn virus.

**Response to the reviewer:** Thank you for your comment. We initially did not get it correct, but the entire paragraph has been revised and accurately restated (Line 62-69)

3. Line 62/63: The sentence: ‘The virus belongs to the Filoviridae family, which is the same family as the Ebola virus’ is wrong. Ebola virus is one of the orthoebolaviruses and is the representative virus of the species Orthoebolavirus zairense. This sentence needs correct nomenclature as well as English language editing.

**Response to the reviewer:** We thank the reviewer for pointing out this. The sentence has been rephrased entirely and the appropriate nomenclature has been utilized (line 64-66).

4. Line 63/64: The sentence ‘Fruit bats (Rousettus aegyptiacus) are widely believed to be the reservoirs of Marburg viruses’. It has been established that R. aegyptiacus are reservoirs of orthomarburgviruses, so it is incorrect to state that they are widely believed to be reservoirs

**Response to the reviewer:** We appreciate the reviewer's observation. We have rephrased our sentence as suggested and we have followed the instruction of how the virus should be written as per new taxonomy rule (Line 68-69).

5. Line 77/78: ‘Since EVD and MVD belong to the same family, they share common symptoms and are both transmitted through contact.’ This sentence implies that all viruses belonging to the family Filoviridae share common symptoms. But this is not the case. It would be better to rephrase the sentence to just state that EVD and MVD have common symptoms. Also add a citation for this.

**Response to the reviewer:** We appreciate the comment. The sentence has been re written in and citation added (Line 82-83).

6. Line 161: Will benefit from English language editing.

**Response to the reviewer:** We appreciate the reviewer's comment. We have made edits to the sentence (Line 166-167).

7. Line 390: the term SUDV was not replaced by EVD. What is in the manuscript is ‘Ebola’ and not EVD.

**Response to the reviewer:** We thank the reviewer for pointing out this. We have replaced the term Ebola with SVD throughout the entire manuscript to ensure consistency (line 393).

**Reviewer No. 2**

**Comments/concerns/suggestions by reviewer 2:**

1. line 36 (abstract): spell out RT-PCR.

**Response to the reviewer:** We thank the reviewer for the observation. RT-PCR has been spelled out (Line 36).

2. Line 37: instead of "physically followed up" consider followed up in-person

**Response to the reviewer:** Thank you so much for the suggestion. The term physically followed up has been changed to followed up in person (Line 37).

3. Line 43: consider also providing median age as there are extremes in the age range

**Response to the reviewer:** We thank the reviewer for the comment. Median age has been used instead of mean (Line 43-44).

4. Line 62: consider deleting "and non-human primates" as this list is not exhaustive.

**Response to the reviewer:** Thank you, the sentence containing "non-human primate" has been omitted and the whole sentence has been rephrased (Line 62-64).

5. Line 79: The outbreak in Uganda was due to Sudan virus, so use Sudan virus disease instead of EVD. EVD now only refers to disease due to Ebola virus (species Orthoebolavirus zairense)

**Response to the reviewer:** We are grateful to the reviewer for their comment. After fully comprehending the new taxonomy rule, we have updated the outbreak from EVD to SVD (Line 77-89).

6. Line 82: viral hemorrhagic fever should not be capitalized

**Response to the reviewer:** We are grateful for the observation. Viral hemorrhagic fever has been re written in small letters (Line 87).

7. Line 120: for clarity, consider cutting "having any of the following"

**Response to the reviewer:** Thank you for your comment. The line that said "having any of the following" has been deleted (Line 125).

8. Line 137: If you decide to use orthoebolaviruses instead of EVD, considering using the term marburg virus instead of MVD so that you are listing viruses

**Response to the reviewer:** Thank you for your suggestion. The sentence has been rewritten to incorporate your suggestions (Line 141-142).

9. Line 197: consider providing median age (as comment above).

**Response to the reviewer:** We appreciate the reviewer's suggestions. Median age has been used in all area as suggested (Line 200)

10. Line 225: clarify what you mean by "without any expert supervision" ...i assume this means without IPC supervision?

**Response to the reviewer:** We thank the reviewer for the comment. We have revised our sentence to clearly indicate that the individuals were not supervised by health professionals trained on IPC (Line 227-229).

11. Line 369: malaria and typhoid fever should not be capitalized

**Response to the reviewer:** We thank the reviewer for the comment. Malaria and Typhoid have been re written in small letters (Line 372).

12. Line 371: please note that pauci-symptomatic marburg virus disease has been detected in Uganda during follow up serosurveys. Thus it is possible there were more people infected, but were not detected during the outbreak. This is especially important given that Marburg virus can persist in the semen of male survivors"

**Response to the reviewer:** Thank you for your comment. We have added more information in our sentence to incorporate your suggestions (Line 376-377).

13. Line 296 – 301: For case 8, what was the date of the positive Marburg test? What was the date of admission to the MTU?

**Response to the reviewer:** We thank the reviewer for the comment. Date the case tested positive and date of admission has been added (line 303-304).

14. Line 302 – 309: For case 9, it does not seem that she could have been infected by case 1, 4, or 8.

-Assuming an incubation period of 2-21 days and assuming her illness onset date is correct, she was exposed to Marburg virus 24 march – 6 april

-Case 1 died March 1

-Case 4 died March 16

-Case 8 was admitted to the hospital on March 21 and died April 10. Assuming case 8 was admitted to the MTU on March 21 and isolated, the Case 9 (mother of case 8) should not have had any contact with case 8 after he was admitted to the MTU?

**Response to the reviewer:** We thank the reviewer for the comment. We have revised our paragraph to make it clearer that at first she was contact of case 1 and 4, we have omitted issues of most likely exposure. Additionally, we have improved the sentence to indicate that the mother was the primary caregiver for her son in MTU due to his age (Line 306-310).
